# The efficacy of pivmecillinam in oral step-down treatment in hospitalised patients with *E. coli* bacteremic urinary tract infection; a single-arm, uncontrolled treatment study

**DOI:** 10.1186/s12879-022-07463-7

**Published:** 2022-05-19

**Authors:** Bjørn Åsheim Hansen, Nils Grude, Morten Lindbæk, Tore Stenstad

**Affiliations:** 1 Department of Infectious Diseases, Vestfold Hospital Trust, Postbox 2168, 3103 Tønsberg, Norway; 2 Department of Microbiology, Vestfold Hospital Trust, Postbox 2168, 3103 Tønsberg, Norway; 3 The Antibiotic Centre of Primary Care, Department of General Practice, Institute of Health and Society, University of Oslo, 0317 Oslo, Norway

**Keywords:** Pyelonephritis, Urosepsis, Pivmecillinam, Mecillinam, Oral treatment, Beta-lactam, PROM, Hospitalised

## Abstract

**Background:**

The role of oral beta-lactam antibiotics in treating febrile urinary tract infections (UTI) is not yet definite. Today, fluoroquinolones together with trimethoprim–sulfamethoxazole (TMP–MTX) are considered standard of care and often the only available evidence-based oral treatment for febrile UTI. This study clarifies the efficacy and safety of pivmecillinam (PIV) used as step-down therapy for bacteremic urinary tract infection (UTI).

**Methods:**

A single-arm, uncontrolled treatment trial was conducted in the period September 2017–March 2020. Candidates for inclusion were men and women suffering from *E. coli* bacteremia due to UTI and were consecutively included in a Norwegian hospital. Exclusion criteria were among others: other ongoing bacterial infection, septic shock, pyonephrosis/abscess and pregnancy. After 3 days of parenteral antibiotic, the treatment was converted to the study drug; oral PIV 400 mg QID for 1 week. Primary endpoint was a combination of three elements; afebrility, no need for retreatment and improvement in self-reported health status. Test Of Cure (TOC) was 1 week post-treatment. Secondary endpoints included among others microbiological efficacy and CRP value < 30 mg/L.

**Results:**

Of 476 screened subjects, 53 patients were included. Median age was 67 years, 28 (56%) were women. 50 patients were evaluated for per-protocol analysis. 44 of 50 patients (88%) (95% CI [75.7–95.5]) reached the primary endpoint on TOC. 14 of 48 patients (29.2%) had significant growth (> 10^3^ CFU/mL) of *E.coli* on TOC. CRP-level was strongly associated to treatment outcome, (OR 0.006 [95% CI 0.00–0.11], p < 0.001).

**Conclusions:**

This trial documents that PIV 400 mg QID given for 1 week following 3 days of parenteral antibiotics, is a suitable treatment option in patients suffering from bacteremic UTI due to *E. coli*. Randomised clinical trials studying the efficacy of PIV vs standard of care of febrile UTI are warranted.

*Trial registration* The trial was registered at ClinicalTrials.gov under the identifier: NCT03282006 13/09/2017 and approved by The Regional Committees for Medical Research Ethics South East Norway (2015/2384/REK sør-øst) and the Norwegian Medicines Agency (SLV; reference No 16/06018-09; EudraCT No 2016-000984-18) before initiation

**Supplementary Information:**

The online version contains supplementary material available at 10.1186/s12879-022-07463-7.

## Background

The role of oral beta-lactam antibiotics in treating febrile urinary tract infections (UTI) is not yet definite. Today, fluoroquinolones together with trimethoprim–sulfamethoxazole (TMP–MTX) are considered standard of care and often the only available evidence-based oral treatment for febrile UTI [1, 2]. Due to risk of potentially permanent side effects and concerns regarding increasing resistance, the use of fluoroquinolones should be restricted [3, 4]. Use of TMP–MTX is challenged by limited microbiological susceptibility, drug intolerance and side-effects [5, 6]. Hence, finding oral alternatives in treating febrile UTI is crucial.

One potential option for treating febrile UTI is mecillinam—an antimicrobial agent from the amidinopenicillin group with effect substantially against gram-negative microbes [7–9]. Orally formulated, the drug is chemically modified with pivalic acid to pivmecillinam (PIV) for better bioavailability [10]. The mechanism of action is exercised by inhibition of penicillin-binding protein 2 and the effect is bactericidal. The drug has high renal tissue concentrations compared to serum [11], it is safe and well tolerated [12, 13]. In the Scandinavian countries mecillinam has been distributed for decades, and the resistance rates in the community are still low (~ 5%) [5, 14, 15].

While the evidence for using PIV in treating lower UTI is solid [12, 16–18] the documentation in treating febrile UTI, is sparse. In 2018, a literature review studying the efficacy of PIV in treating acute pyelonephritis and bacteremia, recommended the drug to be considered as a treatment option, but the report emphasized the need for further clinical research and documentation [19].

The aim of this study was to clarify the efficacy and safety of PIV in consecutively hospitalised patients as oral step-down treatment after initial parenteral antibiotic for bacteremic UTI.

## Methods

This project was a prospective, single-arm, uncontrolled treatment study where the participants were consecutively included in a Norwegian hospital.

### Approval, ethics and funding

The trial was registered at ClinicalTrials.gov under the identifier: NCT03282006 13/09/2017 and approved by The Regional Committees for Medical Research Ethics South East Norway (2015/2384/REK sør-øst) and the Norwegian Medicines Agency (SLV; reference No 16/06018-09; EudraCT No 2016-000984-18) before initiation. The Clinical Trial Unit at Oslo University Hospital monitored the project. All participants gave written informed consent according to the International Conference on Harmonization of Good Clinical Practice and the Helsinki Declaration.

### Participants

Patients ≥ 18 years old hospitalised at Vestfold Hospital Trust from September 2017 through March 2020 with *E. coli* bacteremia due to febrile UTI were eligible for inclusion. Both men and women were recruited. Inclusion was performed during the first 3 days after hospital admission. Inclusion/exclusion criteria are summarized in Table 1.

**Table 1:** Eligibility and exclusion criteria in the study

Eligibility criteria
*E. coli* bacteremia
≥ 18 years old
Relevant evidence of UTI^#^
Exclusion criteria
Evidence of other ongoing bacterial infection
Septic shock and treatment with vasopressors due to organ failure
Pyonephrosis or perinephric abscess
Mecillinam-allergy
* E. coli* isolate resistant to mecillinam (MIC > 8 mg/L)
Hereditary organic acidurias
Other parenteral pretreatment than standard of care^##^
Severe renal failure (e-GFR < 15 mL/min)
Pregnancy/breast feeding
Severe neutropenia (< 0.5 × 10^9^ cells/L)
Obvious symptoms of prostatitis
Concurrent valproate treatment
Treatment with other gram-negative antibiotic
Previous study participation
Lack of co-operability^###^

*e-GFR* estimated glomerular filtration rate

^#^Relevant symptoms of UTI would be dysuria, pollakisuria, suprapubic pain or costovertebral tenderness. Relevant microbiological finding would be quantitative urine cultivation (≥ 10^3^ CFU/mL) of *E. coli* showing identical susceptibility pattern as the isolate from blood culture. Relevant urine-analysis were positive leukocyte esterase- or nitrite-test on dipstick

^##^Ampicillin, gentamicin, cefotaxime, piperacillin/tazobactam

^###^Confusion, mental retardation or terminally ill cancer-patients

### Interventions, microbiology and patient reported outcome

**Fig. 1:**
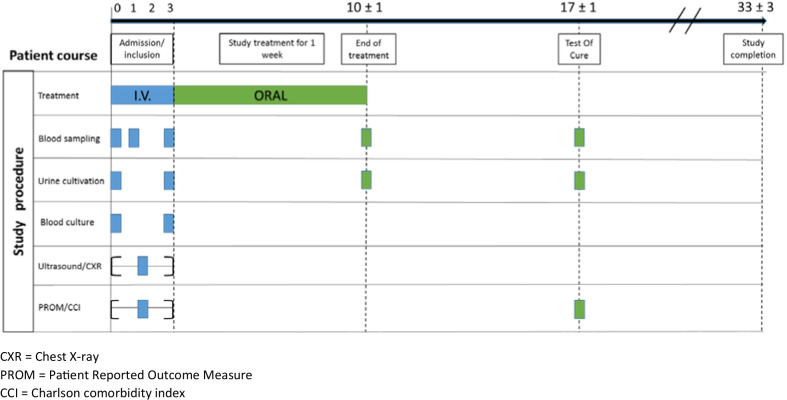
Patient course from admission to study completion

An overview of the treatment intervention and patient course is summarized in Fig. 1. A variety of blood samples, including blood cultures, and midstream urinary sample or secondarily a catheter sample, for quantitative urine culture, were collected at hospitalisation (day 0) and on discharge from hospital (day 3). Ultrasonic examination or CT-scan of the urinary tract was performed prior to inclusion and chest X-ray within the 1st day. Bacterial isolates from blood and urine were identified by Matrix Assisted Laser Desorption Time of Flight Mass Spectrometry (MALDI-TOF, Bruker Daltonics) and tested for antibiotic susceptibility according to European Committee on Antimicrobial Suseptibility Testing (EUCAST) disc diffusion methodology [20]. Patients infected with fully susceptible isolates (MIC ≤ 1 mg/L) and non-wild-type *E. coli* isolates with reduced susceptibility to mecillinam (MIC = 2–8 mg/L) were considered candidates for inclusion. Patients with resistant isolates (MIC > 8 mg/L) were not included. Extended-spectrum beta-lactamases (ESBL) producing isolates did not disqualify from study participation. All bacterial isolates from blood cultures were frozen for further research. All participants were scored according to the Charlson Comorbidity Index (CCI) [21]. Self-rated health status was measured using three different methods, Table 2. To compare values in the EQ-5D-3L health state, we used the Danish TTO set of weights [22]. The protocol describes the use of EQ-5D-3L combined with EQ-VAS-thermometer as PROM (Patient Reported Outcome Measure). In designing the CRF (Case Report Form) we added a standardised questionnaire as a third method for quantifying self-rated health status.

**Table 2:** Methods for evaluating self-experienced health status

Method	Characteristics
EQ-5D-3L questionnaire	Five-dimensional descriptive questionnaire comprising: mobility, self-care, usual activities, pain/discomfort and anxiety/depression. Each dimension has three levels: no problems, some problems and extreme problems
EQ VAS-thermometer	A vertical visual analogue scale where the endpoints are labelled “best imaginable health state” and “worst imaginable health state”
Question asked by study personal	Single question asked on TOC: “How is your general condition today compared to when you left the hospital: “unchanged”, “better” or “worse”?”

*TOC* Test Of Cure

### Antibiotic treatment and follow-up

The participants were initially given parenteral antibiotics (ampicillin/gentamicin, cefotaxime or piperacillin/tazobactam) according to Norwegian guidelines [23]. Parenteral drug administration was ended on day 3 and the participants were put on oral treatment with investigational drug (PIV) tablets 400 mg (Selexid, LEO-pharma A/S) QID. In case of fever on day 3, the patients were observed in hospital for an additional 24 h without deviation from the study protocol in other respects. The patients received study medication in a pre-filled cassette from the department and the duration of the treatment was 1 week.

Three days after discharge, the patients were contacted by phone by the study personnel (trained nurse or doctor) and queried for adverse events or treatment failure. One week after discharge, the patients delivered blood samples and urine specimen and were interviewed by phone. The test of cure (TOC) clinical assessment was carried out 2 weeks ± 1 day following discharge; i.e. 1 week after treatment cessation. PROMs were performed as outlined above together with blood pressure, pulse and temporal body temperature recordings (Exergen Thermometer TemporalScanner Model TAT-5000), as well as bladder scan for residual urine assessment (BladderScan BVI 3000). A urine specimen was collected for bacteriology. Blood tests were sampled and the medication cassette was compiled and checked regarding compliance.

The patient file was checked 1 month ± 3 days following discharge to document whether the patient was still alive or readmitted to hospital (due to urinary tract infection, Clostridioides difficile Infection (CDI) or other causes). The participant was contacted to clarify whether urinary tract infection or CDI had occurred since TOC.

### Outcome measures

The primary endpoint was defined as a combination of three elements; afebrility (temporal body temperature < 38 °C), no need for retreatment and improvement in self-reported health status at TOC. In the assessment of treatment success, all three aspects were weighted equally and all three criteria had to be redeemed.

Secondary endpoints included non-significant bacterial growth in urine on TOC, C-reactive protein value < 30 mg/L, treatment-requiring UTI or readmission due to UTI after TOC but less than 1 month after discharge, readmission for other reasons or death less than 1 month after discharge, serious adverse drug events and occurrence and CDI.

### Safety measures

Interim analysis was not done, but certain rules for premature study-abortion were established in case of absence of clinical cure on day 10 or need for hospital admission due to SAE or treatment failure for a significant share of patients as described elsewhere (www.ClinicalTrials.gov).

### Statistics

Confidence interval (CI) for the observed proportion of success of primary outcome was obtained using binomial exact 95% confidence interval. To analyse potential effect of different variables on probability of success of the treatment, we performed univariate logistic regression and reported odds ratios, 95% CIs and p-values for CRP-values, age, sex, persistent *E. coli* bacteriuria, IDC (indwelling urinary catheter), presence of non-wild type *E. coli*, CCI, renal function and diabetes mellitus. Data was analysed using the SPSS Statistics 26 package and Stata.

## Results

### Study population

**Fig. 2:**
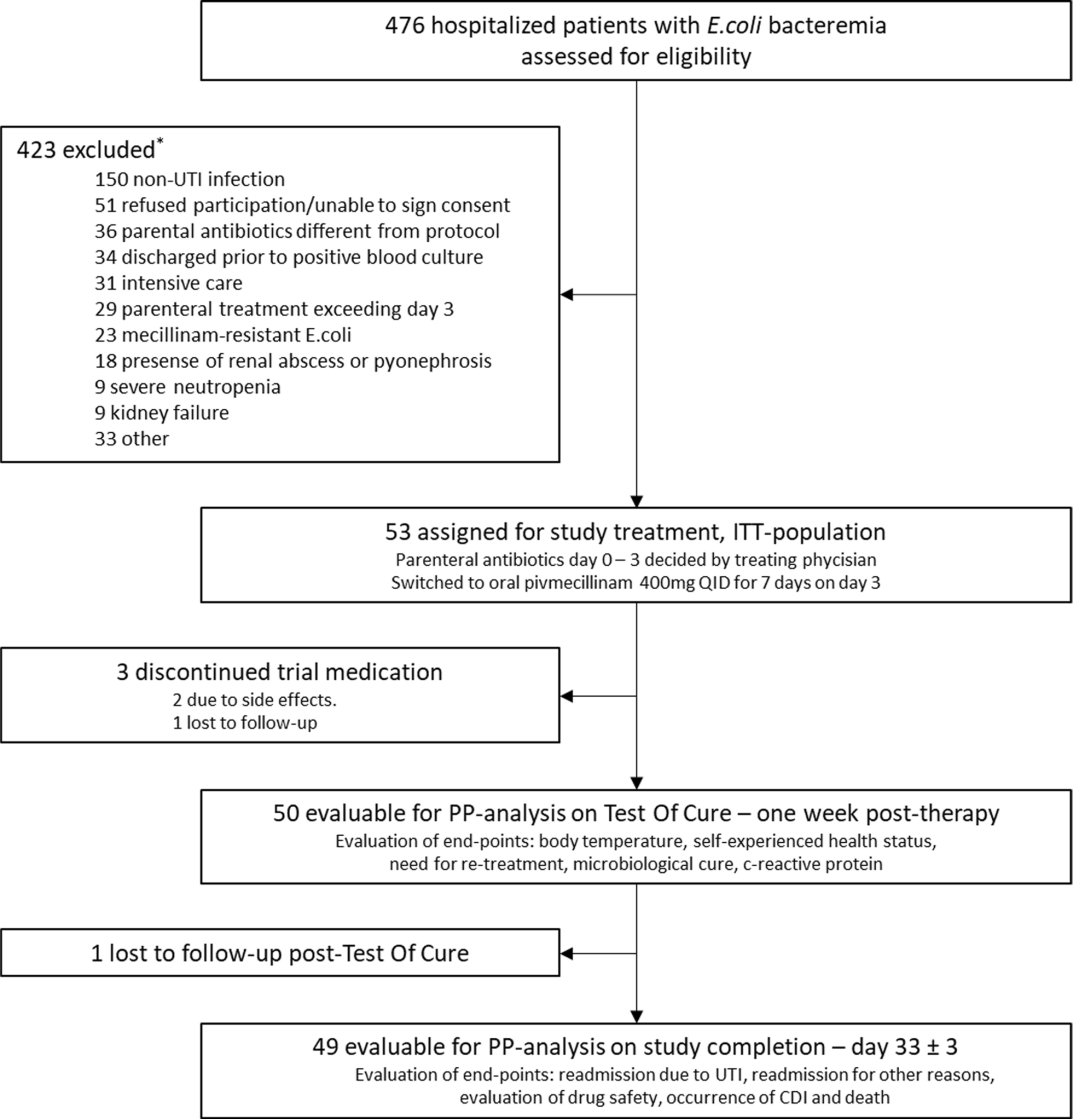
Trial algorithm

The trial algorithm, including the number of patients assigned for study treatment, is visualised in Fig. 2. The main reason for exclusion was a different origin of infection, e.g. cholecystitis, cholangitis, diverticulitis, liver abscess or synchronous bacterial infection outside the urinary tract.

Fifty-three patients initiated treatment with the investigational drug. Two patients interrupted the treatment due to adverse events and one refused to continue trial participation without reason. One participant was diagnosed with concomitant viral pneumonia (respiratory syncytial virus) and on discharge referred to a nursing home. After 3 days, the patient was readmitted to the hospital due to fever, dyspnea and clinical deterioration. Broad-spectrum intravenous antibiotics were administered, classifying the participant as treatment failure. The patient was not able to adhere to the following study protocol but was included among the 50 patients evaluated for primary endpoint. Seven participants had malignant comorbidity and one had solid organ transplant (pancreatic allograft). Characteristics of the participants are listed in Table 3.

**Table 3:** Clinical and microbiological characteristics at baseline of 50 patients with *E. coli* bacteremic UTI—Per-protocol population (n = 50)

Baseline characteristics	Result
Age, median (range), y	67 (34–87)
Women	28 (56.0)
Charlson comorbidity index—CCI, mean	1.8
CCI < 3	39 (73.6)
CCI > 5	4 (7.5)
Diabetes mellitus	11 (22.0)
Cancer (lymphoma/leukemia/solid tumour)	7 (13.2)
Indwelling urinary catheter	8 (15.1)
BMI^a^, median (range)	26.4 (16.6–38.1)
Positive blood culture on admission	50 (100.0)
Positive blood culture on day 3	1 (2.0)
*E. coli* as causative agent	50 (100.0)
Parenteral antibiotic day 0–3	
Ampicillin + gentamicin	27 (54.0)
Cefotaxime	7 (14.0)
Piperacillin/tazobactam	0 (0.0)
Ampicillin	1 (2.0)
Combination of above	15 (30.0)
Q-SOFA^b^ ≥ 2 on admission	2 (4.0)
SIRS^c^ ≥ 3 on admission	31 (62.0)
C-reactive protein on admission, mg/L, median (range)	163 (1.8–349.0)
Procalcitonin on admission, mcg/L, median (range)	1.0 (< 0.1–56.1)
White cell count on admission, 10^9^ × cells/L, median (range)	14.5 (4.7–137.0)
Neutrophils on admission, 10^9^ × cells/L, median (range)	11.7 (1.9–23.1)
E-GFR^d^ on admission, median (range)	59.5 (23–107)
Body temperature ≥ 39.0 °C	20 (40.0)
Symptoms of UTI on admission^e^	29 (58.0)

Microbiology	Result
Positive urine culture on admission (*E. coli* > 10^3^ CFU^f^/mL)	45 (90.0)
Non-wild type (mecillinam inhibition zone 16–19 mm)	4 (8.0)
ESBL^g^-producing	2 (4.0)
Resistant to ciprofloxacin	6 (12.0)
Resistant to TMP–SMX^h^	11 (22.0)
Resistant to gentamicin	1 (2.0)
Resistant to cefotaxime	2 (4.0)
Resistant to ampicillin	18 (36.0)
Resistant to meropenem	0 (0.0)
Resistant to piperacillin/tazobactam	3 (6.0)

Data are number (%), unless otherwise indicated

^a^Body Mass Index, ^b^Quick Sepsis-related Organ Failure Assessment, ^c^Systemic Inflammatory Response Syndrome, ^d^Estimated Glomerular Filtration Rate, ^e^One of the following: dysuria, pollakisuria, hematuria, suprapubic pain or abdominal pain, ^f^Colony Forming Units, ^g^Exstended Spectrum Beta-Lactamases, ^h^Trimethoprim sulfamethoxazole

### Microbiological characteristics

All of the 476 subjects screened for eligibility had bacteremic growth of *E. coli*, of whom 23 (4.8%) were infected with mecillinam-resistant strains which corresponds to national susceptibility data [5]. Microbiological characteristics of the participants’ *E. coli* isolates are summarised in Table 3. Two isolates were expressing ESBL. Forty-five subjects (90%) had growth of *E. coli* in their urine on admission. The main reason for urine culture failure were pre-sampling antibiotics (three patients). Two patients did not deliver a urine sample.

### Primary outcome—clinical cure

Of the 50 patients included in the per-protocol evaluation, 44 patients achieved success in the primary composite endpoint (Table 4). None of the patients had fever at TOC (missing data = 1). Four of the patients were readmitted to hospital, of whom two patients were diagnosed with UTI, one with spondylodiscitis and one with calculous cholecystitis. In total five patients needed retreatment. The results from the three different methods measuring patients reported health status, showed considerable degree of intrapersonal divergence and are listed in Additional file 1: Fig. S1. Characteristics of the six patients with treatment failure are summarised in Table 5.

**Table 4:** Outcome measures at Test Of Cure—TOC day 17 ± 1 and on study completion day 33 ± 3

Primary outcome—TOC—day 17 ± 1	n/N	%
Clinical efficacy^1^		
Afebrile	49/49	100.0
No need for re-treatment	45/50	90.0
Improved health status	46/49	93.9
**Composite primary end-point (all three above)**	**44/50**	**88.0** **95% CI [75.7–95.5]**
Secondary outcome—TOC—day 17 ± 1		
Bacteriologic result—urine culture^2^		
No growth (< 10^3^ CFU^*^/mL)	19/48	39.6
* E. coli* 10^3^ → 10^5^ CFU/mL	14/48	29.2
Other uropathogen	2/48	4.2
Mixed flora/contaminated	13/48	27.1
C-reactive protein (mg/L)^2^		
< 30	43/48	89.6
≥ 30	5/48	10.4
Secondary outcome measures—day 33 ± 3		
Readmission due to UTI < 1 month after discharge	4/50	8.0
Including 2 patients readmitted prior to TOC		
Including 1 patient treated with elective transurethral bladder biopsy		
Readmission for other reasons < 1 month after discharge	4/50	8.0
Calculous cholecystitis = 1		
Spondylodiscitis = 1		
Rectal hemorrhage = 1		
Observation for abdominal pain = 1		
Treatment-requiring UTI < 1 month after discharge	6/50	12.0
Death < 1 month after discharge	0/50	0.0
Occurrence of CDI^3^	1/50	2.0

Per-protocol population

^1^Body temperature and patient reported outcome measure not done in one patient

^2^Urine and blood sample not done in two patients

^3^*Clostridioides Difficile* Infection

^*^Colony Forming Unit

**Table 5:** Characteristics of patients who failed to reach clinical cure on TOC

Pat. Index	Age (y)	Sex (m/f)	CCI	IDC^4^	PCT mcg/L			CRP mg/L			Lkc 10^9^ × cells/L	Neutrophils 10^9^ × cells/L	Urine culture^3^		Mecillinam Inhibition-Zone, mm	Ultrasound	Primary end-point			Compliance, skipped doses
					Max^1^	Day 10	Day 17	Max^1^	Day 10	Day 17	Day 17	Day 17	Day 10	Day 17			Body temp	Health-status	Re-treatment	
10	66	m	2	No	11.5	< 0.10	< 0.10	280	66	169	109^2^	3.4	a	a	26	Normal	36.4	Better	Yes	0
11	62	f	1	No	13.9	< 0.10	< 0.10	225	100	36	8.4	5.0	a	d	23	Normal	37.4	Better	yes	0
27	72	m	0	Yes	17.9	0.20	0.20	315	25	159	6.5	4.6	a	a	27	Normal	37.7	Worse	Yes	0
30	72	f	2	No	–^5^	0.20	–	527	62	–	–	–	–	–	19	Normal	–	–	Yes	–
38	58	f	2	No	0.80	< 0.10	< 0.10	283	5	124	10.6	6.8	d	b	25	Normal	37.6	Worse	Yes	0
45	63	m	1	Yes	1.50	–	< 0.10	157	13	7	8.5	5.6	a	b	26	Normal	37.1	Worse	No	0

Study treatment pivmecillinam 400 mg QID for 1 week (n = 6)

*CCI* Charlson Comorbidity Index

^1^Maximum value during admission

^2^Chronic lymphatic leukemia

^3^a =  < 1.000 CFU/mL, b = *E. coli* > 100.000 CFU/mL, c = Other UT pathogen, d = mixed flora/contaminated

^4^Indwelling urinary catheter

^5^Not documented

### Secondary outcomes

On TOC, 14 out of the 48 patients (29.2%) had significant growth (> 10^3^ CFU/mL) of *E. coli* (Table 2). In 12 cases, the isolates exposed the same susceptibility pattern as in the pre-treatment cultivation.

The treatment showed high efficacy with respect to biochemical outcome measures with normalisation of inflammatory markers as total amount of leukocytes, neutrophils, CRP and PCT in 90% of the patients. Five patients had CRP ≥ 30 mg/L on TOC. Among these, four were treatment failures giving a significant lower probability of treatment success on TOC for CRP ≥ 30 mg/L compared to CRP < 30 mg/L (p < 0.001; OR = 0.006, 95% CI 0.00–0.11). The associations between clinical/laboratory parameters vs primary outcome are listed in Additional file 1: Table S2.

At study completion (day 33 ± 3), six patients had received antimicrobial treatment for UTI after TOC. One patient developed CDI 2 weeks after treatment completion following intravenous cephalosporin treatment at readmission due to respiratory infection as described above.

### Adverse events

In general, the study drug was well tolerated. The side effects were essentially mild without need for further intervention or treatment. The exception was for two patients who developed intolerable side effects (skin-eruption and nausea/vomiting) which resulted in interruption of the study protocol. An overview of adverse events is displayed in Additional file 1: Table S3. The participants who completed the trial were largely compliant to the study treatment and no patient skipped more than two tablets. Forty-two participants (84%) adhered completely to the treatment regimen.

## Discussion

This single-arm, uncontrolled treatment study with 50 patients with bacteremic UTI due to *E. coli*, documents that PIV 400 mg QID given for 1 week following three days of parenteral antibiotics, is a responsible treatment option. Forty-four of 50 patients (88%), [95% CI 75.7–95.5] achieved clinical cure on TOC. The treatment was safe and well tolerated—two out of 53 patients (3.8%) interrupted the treatment due to side effects. Of the 50 per-protocol participants, one developed CDI 2 weeks after cessation of PIV-therapy and subsequent cefotaxime therapy.

Based on the 48 of 50 patients who supplied urine culture on TOC, the bacteriological cure rate was 70.8%—however, 13 samples showed growth of mixed flora interpreted as contamination. Eleven (22%) out of the 50 patients required retreatment for UTI within the 1st month. Five patients were retreated prior to TOC and six post TOC. The reasons for retreatment in the latter are not illuminated, and whether these incidents were relapses or reinfections are unknown.

Of our patients suffering from bacteremic UTI, only 58% reported classical UTI symptoms, emphasizing the importance of not ruling out the urinary tract as the focus of infection in case of modest symptoms.

The number of patients in this study is somewhat low to perform multivariate analysis, thus only cross tables were used to look for association of treatment success by a variety of patient characteristics: sex, occurrence of non-wild-type *E. coli*, indwelling urinary catheter, Charlson comorbidity index, renal function and diabetes mellitus. No significant association were uncovered. However—CRP < > 30 mg/L on TOC showed strong association to clinical outcome (p < 0.001).

Our results support the previous sparse documentation indicating that PIV should be taken into consideration when it comes to treating febrile UTI. All participants had bacteremic growth on inclusion, implying the potency of PIV therapy for urinary parenchymal infections after initial parenteral treatment. Several studies describe lower clinical cure rate in patients treated with “oral β-lactams” [24–27] and it has been postulated that the observed inferiority of beta-lactam antibiotics in treatment of invasive UTI can be explained by inadequate dosage regime when compared to other antibiotic classes (e.g. flouroquinolones and TMP-SMX) [27–30]. Hence, the treatment regimen in this study was 400 mg QID for 1 week; i.e. high dose PIV. We did not exclude patients infected with non-wild type *E. coli* showing either reduced susceptibility to mecillinam (four patients) or ESBL-production (two patients). Although one patient infected with non-wild type *E. coli* failed to reach clinical cure, we still believe high-dosed PIV is an adequate treatment given that the strains show an inhibition zone ≥ 16 mm. According to the SPC, the recommended maximum dose of PIV is 400 mg TID, allowing a serum mecillinam concentration above wild type *E. coli* MIC approximately 38% of the time. As the present study also included patients with intermediate susceptibility *E. coli* infection, time above MIC would only be 25% of the time using standard dose, in contrast to approximately 33% with the QID dosing regimen. For this reason, the fact that isolates expressing ESBL were included and considering a heterogeneous, bacteremic population, the high dose regimen was chosen.

We present results from a treatment study that includes patients that often are omitted—elderly and patients with comorbidities. The trial included patients with i.e. malignancy, renal failure, cardiovascular disease, organ transplantation, diabetes mellitus, neurological deficiencies and indwelling catheter. The study profile and patient care did not deviate significantly from everyday hospital practice. This, combined with the proportion of male subjects and the fact that every patient had bacteremic infection—strengthens the applicability of our results.

The effect contributed by the parenteral treatment has not been studied in this trial. The combination of ampicillin/aminoglycosides was the most frequent treatment given and the main reason for change in parenteral antimicrobial regime was reduced renal function. Whether the outcome can be entirely explained from this treatment cannot be ruled out, but appears less probable in perspective of the recommendations for treatment duration of bacteremic infections. We assume that patients referred to hospital with suspected bacterial infection will still be put on empirical intravenous treatment and our study suggest that conversion to oral PIV is safe given fulfilment of this trial’s eligibility criteria.

In designing the study, we decided to include an evaluation of self-experienced health status in the primary end-point. In the absence of any disease-specific evaluation form, we concluded on using three different tools as described above. However, the results showed significant divergence. In the interpretation of the results, we concluded that health status was “Improved” when the patient reported improvement in one of the three methods. In retrospect, we assume that generic PROM such as EQ-5D-3L is difficult to use individually in patients suffering from febrile UTI. Regarding fever—none of the patients were febrile on TOC—suggesting that body temperature is not sensitive enough as an outcome measure. Need for retreatment appear to be a reasonable parameter—hence it absorbs clinical, biochemical and microbiological interpretation. Retrospectively, it can be argued that emphasising all three parameters equally, was unfavourable.

Due to insufficient resources, the trial was outlined without a control-group but rather to be a non-inferiority study where the comparator was an estimated efficacy of standard of care according to Norwegian guidelines. Working with the trial, we recognized difficulties in the comparison of efficacy due to differences in outcome measures. The study design was then converted to a single-arm, uncontrolled treatment trial with no comparator.

## Conclusion

This trial documents that PIV 400 mg QID given for 1 week following 3 days of parenteral antibiotics, is a suitable treatment option in patients suffering from bacteremic UTI due to *E. coli*. As suggested by other authors [19], the efficacy of PIV vs standard of care treatment of febrile UTI, should be clarified by randomised clinical trials.

In evaluating clinical curation in future studies, we suggest not using self-experienced morbidity until there exists a validated disease-specific PROM. CRP-level seems to be, however, strongly associated to treatment outcome.

## Supplementary Information

**Additional file 1: Table S1.** Number of subjects screened/excluded/included and reason for exclusion. **Figure S1. **Venn diagram showing variations in self-reported improvement in health status. **Table S2.** Univariate analysis of various clinical and laboratory parameters vs treatment success. **Table S3**. Rates of Adverse Events (AE)/Serious Adverse Event (SAE) among patients treated with pivmecillinam 400 mg QID for 1 week (ITT, n = 53).

## Data Availability

The datasets used and/or analysed during the current study are available from the repository www.synapse.org (https://doi.org/10.7303/syn29042871). Additional datasets are available from the corresponding author on reasonable request.

## Abbreviations

UTI: Urinary tract infection
TMP–SMX: Trimethoprim–sulfamethoxazole
PIV: Pivmecillinam
MIC: Minimal inhibition concentration
e-GFR: Estimated glomerular filtration rate
*E. coli*: *Escherichia coli*
CCI: Charlson Comorbidity Index
TOC: Test Of Cure
PROM: Patient Reported Outcome Measure
CDI: Clostridioides difficele infection
IDC: Indwelling urinary catheter
QID: Quater In Die = four times a day
Q-SOFA: Quick Sequential Organ Failure Assessment
SIRS: Systemic Inflammatory Response Syndrome
CFU: Colony Forming Units
PCT: Pro-calcitonin
CRP: C-reactive protein
Lkc: Leukocytes
ESBL: Extended-spectrum-beta-lactamases
