# Supplementary material for: The efficacy of pivmecillinam in oral step-down treatment in hospitalised patients with E. coli bacteremic urinary tract infection; a single-arm, uncontrolled treatment study
Source: BMC Infect Dis. 2022 May 19;22:478. doi: 10.1186/s12879-022-07463-7 (PMC9118732; doi:10.1186/s12879-022-07463-7)
Supplement: Supplementary file 1 — Additional file 1: Table S1. Number of subjects screened/excluded/included and reason for exclusion. Figure S1. Venn diagram showing variations in self-reported improvement in health status. Table S2. Univariate analysis of various clinical and laboratory parameters vs treatment success. Table S3. Rates of Adverse Events (AE)/Serious Adverse Event (SAE) among patients treated with pivmecillinam 400 mg QID for 1 week (ITT, n = 53). [file 12879_2022_7463_MOESM1_ESM.docx]

Additional files

**Table S1. Number of subjects screened/excluded/included and reason for exclusion.**

| Reason for exclusion | No. |
| --- | --- |
| Non-UTI infection | 150 |
| Unable to sign consent  Hearing loss = 4  Coma = 2  Confusion/dementia = 17  Terminal phase = 11  Aphasia = 3  Not speaking Norwegian = 3 | 40 |
| Refused participation | 11 |
| Parenteral antibiotics different from protocol | 36 |
| Discharged from the hospital and started on alternative treatment before positive blood culture | 34 |
| Intensive care | 31 |
| Parenteral treatment exceeding day 3 | 29 |
| Isolate resistant to mecillinam | 23 |
| Renal abscess/pyonephrosis | 18 |
| Renal failure, e-GFR < 15 mL/hour | 9 |
| Severe neutropenia <0,5 x 10^9 cells/L | 9 |
| Other:  Pregnancy = 2  Allergy to mecillinam = 1  Previous study participation = 4  MORS = 4  Study personal not present = 15  Patient living remote from hospital = 7 | 33 |
| Total no. excluded | 423 |
| Total no. included | 53 |
| Total no. screened | 476 |

**Figure S1. Venn diagram showing variations in self-reported improvement in health status using three different methods: EQ-5D-3L, EQ VAS-thermometer and Interview. Total no. of patients reporting improvement in at least one method, n=47.**


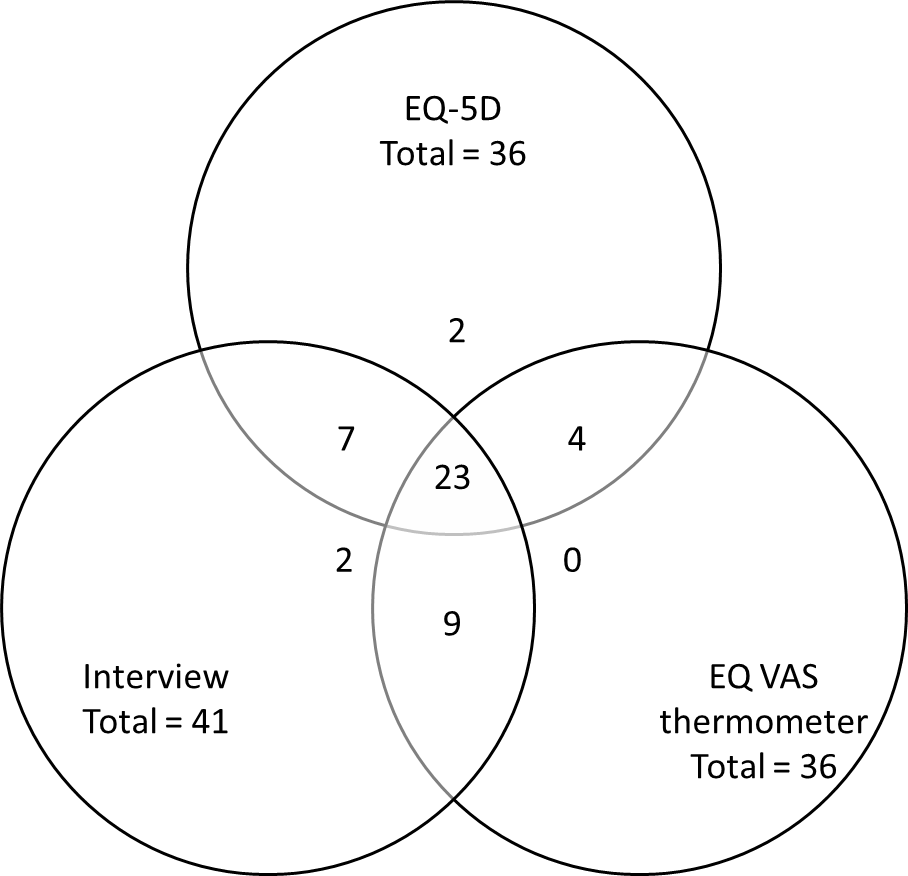


**Table S2. Univariate analysis of various clinical and laboratory parameters vs treatment success.**

| **Clinical or laboratory parameter** | | **n** | **Primary end point result**  **(% of success)** | **ORs**  **(95% CI)** | **P-value** |
| --- | --- | --- | --- | --- | --- |
| Sex |  |  |  |  |  |
|  | Male | 22 | 86.4 | Reference |  |
|  | Female | 28 | 89.3 | 1.32 (0.24-7.26) | 1.000 |
| E.coli bacteriuria on day 17 | |  |  |  |  |
|  | No growth* | 34 | 91.2 | Reference |  |
|  | >1000 CFU | 14 | 85,7 | 0.58 (0.09-3.92) | 0.621 |
| Wild-type E.coli | |  |  |  |  |
|  | Wild-type | 46 | 89.1 | Reference |  |
|  | Non-wild-type | 4 | 75.0 | 0.37 (0.03-4.22) | 0.411 |
| Permanent urinary catheter | |  |  |  |  |
|  | No | 42 | 90.1 | Reference |  |
|  | Yes | 8 | 75.0 | 0.32 (0.05-2.12) | 0.242 |
| C-reactive protein at TOC (day 17) | |  |  |  |  |
|  | <30 mg/l | 43 | 97.7 | Reference |  |
|  | ≥30 mg/l | 5 | 20.0 | 0.006 (0.00-0.11) | <0.001 |
| Extended-spectrum betalactamase | |  |  |  |  |
|  | ESBL - | 48 | 87.5 |  |  |
|  | ESBL + | 2 | 100.0 | - | 1.000 |
| Charlson comorbidity index | |  |  |  |  |
|  | CCI 0-3 | 42 | 85.7 |  |  |
|  | CCI >3 | 8 | 100.0 | - | 0.572 |
| Renal function | |  |  |  |  |
|  | ≥ 60 ml/h | 25 | 88.0 | Reference |  |
|  | < 60 ml/h | 25 | 88.0 | 1.00 (0.18-5.51) | 1.000 |
| Diabetes mellitus | |  |  |  |  |
|  | No | 39 | 87.2 | Reference |  |
|  | Yes | 11 | 90.9 | 1.47 (0.15-14.1) | 1.000 |

ORs: Odds ratios, IC: confidence interval, TOC: Test Of Cure

* or contaminated sample/other pathogen

**Table S3. Rates of Adverse Events (AE)/Serious Adverse Event (SAE) among patients treated with pivmecillinam** **400mg QID for 1 week (ITT, n=53).**

| **Event** | **Result** |
| --- | --- |
| Total no. of events (AE/SAE) | 66 |
| Any digestive | 29 |
| Abdominal pain | 6 |
| Diarrhoea | 9 |
| Nausea/vomiting | 12 |
| Stomatitis | 1 |
| Oesophagitis | 1 |
| Any central nervous system | 18 |
| Headache | 8 |
| Dizziness | 10 |
| Skin eruption/exanthema | 5 |
| Other | 10 |
|  |  |
| Events causing discontinuation of study drug treatment | 2 |
| Skin eruption | 1 |
| Nausea/vomiting | 1 |
|  |  |
| SAE | 4 |
| Readmitted to hospital due to cholecystitis | 1 |
| Readmitted to hospital due to fever/worsened general condition | 1 |
| Readmitted to hospital due to spondylodiscitis | 1 |
| CDI^*^ | 1 |

^*^Clostridioides Difficile Infection
